# Frequency of human papilomavirus and associated factors in gypsy and quilombola women: Human papillomavirus in gypsy and quilombola women

**DOI:** 10.1186/s12905-023-02239-w

**Published:** 2023-04-04

**Authors:** José de Ribamar Ross, Natália Pereira Marinelli, Flavia Castello Branco Vidal, Elamary da Costa Fraga, Maria do Desterro Soares Brandão Nascimento, Marco Aurélio Palazzi Safádi

**Affiliations:** 1 Maranhão State University, Caxias, Maranhão Brazil; 2 Federal University of Piauí, Teresina, Piauí Brazil; 3 Technical School of Teresina, St Dirce Oliveira, Ininga, Teresina, PI 64048-550 Brazil; 4 Medical Sciences, Santa Casa de São Paulo College, São Paulo, Brasil

**Keywords:** Ethnic groups, Group with ancestors from the African continent, Minority groups, Papillomaviridae, Cervical neoplasms, Cervical intraepithelial neoplasia

## Abstract

**Background:**

The prevalence of Human Papillomavirus (HPV) infection in the general population is widely known, however, there are still few studies related to this infection in minority groups, Thus, the objective is to analyze the frequency of human papillomavirus and associated factors in quilombola and gypsy women.

**Methods:**

Cross-sectional research with 145 quilombola and gypsy women from Caxias, Maranhão. Two Pap smear collections were performed and a questionnaire with 46 questions was applied between January, 2020 and March, 2021. Descriptive analysis and Odds Ratio with 95% confidence interval were performed. The research was approved by the ethics committee.

**Results:**

There were 09 cases of atypia. The frequency of human papillomavirus was 41.37%, with a higher risk in quilombolas 55 (91.70%). Multiple infections were prevalent (53%) with high-risk genotypes 21 (35%). Types 16 and 18 together accounted for 42.85% of cases.

**Conclusions:**

The frequency of human papillomavirus infection was higher than those recorded in the Northeast and Brazil, and therefore type 16 predominated. Due to limitations, the virus lineages and sublineages were not evaluated. Quilombola women had a higher rate of infection than gypsies.

## Introduction

Since ancient times anogenital warts have been mentioned as having an infectious cause, and over time the Human Papillomavirus (HPV) has been mentioned as a sexually transmitted etiologic agent causing cervical cancer; however, only in the 1970s did Profesor Harold Zur Hausen demonstrate the existence of HPV genetic material in cervical cancer specimens using recombinant DNA techniques and the use of molecular hybridization. This research was carried out in 1972 from medical references/hypotheses used about warts turning into epithelial cancer. There it was proven that HPV is the primary element of cervical cancer [1].

Incidence and mortality data place uterine cervical neoplasia as one of the leading female cancers on the globe. The least developed countries have the highest burden, with 80% of all cancers worldwide [2–4].

In the year 2018 worldwide, cervical cancer comprised 6.6% among the most common cancers. In the same year deaths from the disease showed a burden of 7.5%. In Brazil the mortality rate per 100,000 women for cervical cancer in the period 2012 to 2016 ranged from 6.86 to 7.18. In the Northeast region, one of the poorest regions in the country, in 2019 the mortality rate was 6.66/100,000. In Maranhão, the Brazilian state with the worst Human Development Index (HDI), the estimated rate was 12.46/100,000 [5–7].

The quilombola population, even with the implantation of social/citizenship rights with the 1988 Constitution, has not been able to strongly change the way of accessing and availability of services, remaining on the margins of the Unified Health System and other public policies inclusions [8].

In 2011, there were 291 gypsy camps, with an estimated half million inhabitants located in 21 Brazilian states, living in extreme poverty. There are currently 2,859 certified quilombola communities, 61% (1,744) of which are in the Northeast Region. The state with the largest number of communities is Bahia (674), followed by Maranhão (597) [9].

Considering the relation of cervical cancer in groups that have a strong vulnerability, we realize that it is exactly in these disadvantaged ethnic groups that there are broad barriers of accessibility to health actions, arising from economic difficulties, insufficient services and cultural issues [10, 11].

This research aims to analyze the frequency of human papillomavirus and associated factors in quilombola and gypsy women.

## Methods

A cross-sectional research, in which exposure and outcome are assessed concurrently at a point in time. Like a snapshot, it produces a prevalence of the offense and raises questions related to the presence of an association and its significance [12]. The research was carried out in Caxias’ city, state of Maranhão. It is a medium-sized municipality located in the east of the state, in the western part of the Northeast, a transition area between the northern and northeastern regions.

**Fig. 1:**
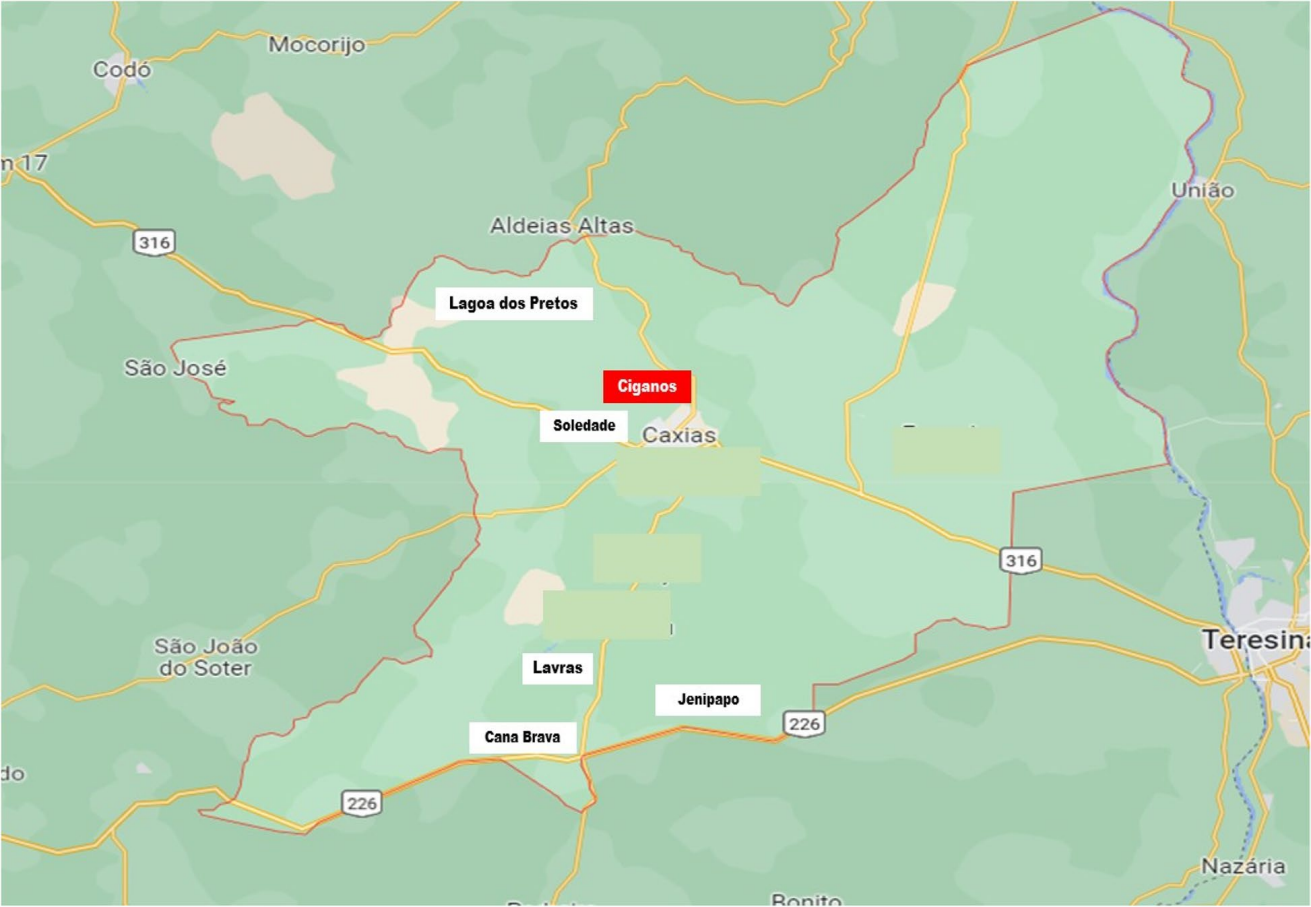
Map of Caxias (MA), Brazil with the spatial distribution of HPV in the 5 quilombola areas and 1 gypsy area. Caxias, MA, Brazil, 2022. Source: Google maps, 2022

The quilombola communities selected in this research were: Cana Brava das Moças, Jenipapo, Lavras Soledade, and Lagoa dos Pretos, all of which are located in the rural area and have difficult access via side roads. Only one gypsy community in the municipality was included. It is in a peri-urban area of the city, called Pé do Morro. The gypsy and quilombola communities are distributed in several districts of the municipality, at extreme points in the north, south and west (Fig. 1). The two complex ethic groups comprise a population of 1,070 inhabitants, 491 men and 579 women. In the age group 10 to 64 years, a population of 331 women was identified, which was the basis for the calculation and definition of the research sample. For the sample calculation we used a 95% confidence interval (5% alpha) to obtain statistical significance. From the population 331 women the sample N calculated by statistician was composed of 145 women who were collected.

Quilombola populations, communities, and peoples in a current context are community groups originating from the old quilombos that originated during the slavery era. Its conception arises as a form of resistance with their implantation in vast communities in rural and urban areas and throughout the country's territorial extension, correlating an ethnic conception. Over time other people have been added to this community in a condition of exclusion.

For the selection of women, the following inclusion criteria were used: women in active sex life; women in the age group of 13 to 64 years and women who do not present any mental or physical condition, be able to collect the Pap smear, in case of a minor having active sex life, and obtain permission from parents or guardians to participate in the collection. The exclusion criteria were: women living outside the locality, outside the age range and clinical conditions that contraindicate the collection of Pap smears, not agreeing to participate in the research and not obtaining parental consent in the case of minors. A questionnaire containing 46 questions related to sociodemographic information, risk factors, and behavioral factors was applied. The collection was carried out in the period from January 18, 2020 to March 16, 2021.

In the collection phase 331 women (quilombolas and gypsies) were recruited. Of the total recruited 201 women presented themselves to the team in the collection phase. After evaluating them and following the exclusion criteria, 56 women were excluded because It presented some condition that contraindicated the collection of the Papanicola exam or were disqualified for some other reason such as not being a resident of the area or for being out of the age range or excessive cervical mucopus.

Four underage women who were between 13 and 17 years old at the time of the research were included in this research. They were identified and special treatment was given to them because there were minors. The mothers were present and consented to their participation, and previous procedures were carried out to orient them about the research and their rights. The minors signed a Free and Informed Consent Form and the mothers signed a Free and Informed Consent Form. Only four (04) minors participated in this research, one 13 years old and three 17 years old.

The selected women were invited to participate in the research and, upon acceptance, signed the Informed Consent Form (ICF). The collection period was from January 18, 2020 to March 16, 2021. The collection period was extended because the quilombos are located in rural and remote areas that are difficult to access. The collection period was extended due to the Covid-19 pandemic, where we had to adopt precautionary health measures, among them: suspension of collection during the peak wave phase of the disease and reduction of the contingent of women participants already in a control phase through vaccination. The impacts of the Covid-19 pandemic on HPV prevalence were not measured in this work. The search for the Pap smear by women was more motivated by the fact that it was performed in the rural quilombola locality that lacks a Basic Health Unit.

Cervical mucosa cells (ectocervix) were collected by exfoliation with a cytobrush®. One brush was collected per patient and stored in tubes of the Kiagen Kit containing a solution for DNA conservation. The DNA extraction tubes were stored in a -4ºC thermal box, -20ºC horizontal freezer and -80ºC vertical freezer in the laboratory.

A second collection of cells from the cervical mucosa was performed in conventional medium with preparation of the smear on slides. The collection of cells from the cervical mucosa started from the ectocervix with the use of the "Ayres" spatula. Next, the material from the ectocervix was spread, being disposed in the horizontal direction. The collection of the endocervix, on its turn, was performed with a cytobrush (cytobrush®), and then the material was spread by rolling the brush from top to bottom in the remaining half of the slide. At the end, the material was fixed with spray. The results of sample suitability and degree of cervical abnormalities were interpreted according to the Bethesda System.

The samples collected were sent for analysis at the Laboratory of the Tumor Biobank and DNA at the Federal University of Maranhão for cytopathological analysis of the slides and molecular biology tests using the PCR (Reverse Chain Polymerase) techniques. In the PCR the primers PGMY 09 and PGMY11 were used in the first round, and in the second round the primers GP + 5 and GP + 6.

The PCR amplified products were sent to the Molecular Biology Laboratory of the Maranhão State University to perform the genetic sequencing of the HPV + DNA identified in the liquid medium samples. The samples positive for HPV DNA in the cervix were typed by sequencing. The determination of HPV genotypes was performed by automated sequencing of the PCR product using the MegaBACE 1000 sequencer.

The variables used were: sociodemographic profile and risk factors related to positivity in cervical cytology tests and DNAHPV + ; the frequency of HPV in women in different cytological results; the frequency of single and multiple HPV infections and, frequency of oncogenic risk HPV. The sociodemographic and risk factor variables were produced by statistical compilation of the collection questionnaire and the HPV frequency variables were extracted from the experimental research methods (Analyses of cervical oncotic cytology, PCR and Gene sequencing).

A structured questionnaire containing 46 questions was applied through individual interviews. The questionnaire was adapted from the model by Batista et al. (2014). The answered questionnaires were implemented in the REDCap software and from these a database was built. In the production of statistical tests REDCap exported its data to SPSS. Initially, the analyses were descriptive and absolute frequency tables and Figures were produced.

The results were discussed in the light of the of the existing literature on the object. The Odds Ratio (OR) was calculated with its 95% CI and the statistical significance level of 5% (*p* < 0.05) to observe the magnitude of the relationship between the variables established.

All experimental protocols were approved by the Research Ethics Committee of the Maranhão State University, with the CAAE number 96368518.4.0000.5554.

## Results

A total of 145 women were included in the research, of which 123(84.83%) were quilombolas and 22 (15.17%) gypsies. The age groups were grouped according to the highest prevalence of infection in these women. Thus, the age group that had the highest number of women was 30 to 50 years, accounting for 56 women (38.62%), followed by the age group of women < 30 years, 49 women (33.79%). Overall, the majority of women were characterized as young and adult, with a frequency of 105 (72.41%). Regarding education, 55 (37.93%) were non-literate and 60 (41.37%) had up to elementary school. As for marital status, 50 (34.48%) were married and 55 (37.93%) were in a stable union.

According to data presented in Table 1, the overall frequency of HPV infection in the sample analyzed was 41.37% (*n* = 60) cases. The overall frequency of cervical cytological atypia in the sample was 6.17% (*n* = 9) cases. In the distribution of atypia cases 4.12% (*n* = 6) women had atypia of undetermined significance (ASC-US and AS-H) and 2.05% (*n* = 03) of women had precursor lesions, classified between NIC I and NIC III (L-SIL and H-SIL) (Table 1).

**Table 1:** Distribution of women positive and negative for HPV according to the degree of cervical atypia in the sample. Caxias, MA, Brasil, 2022

Women from groups	Total	HPV-	HPV +	ASC-US	ASC-H	LSIL	HSIL
		**N %**	**N %**	**N %**	**N %**	**N %**	**N %**
Total	145(100%)	85(58.62)	60(41.37)	04(2.75)	02(1.37)	02(1.37)	01(0.68)

Source: survey data, 2021

*ASC-US* Atypical squamous cells of uncertain significance, *ASC-H* Atypical squamous cells, a high-grade lesion cannot be ruled out, *LSIL* Low-grade Squamous Intraepithelial Lesion, *HSIL* High-grade Squamous Intraepithelial Lesion

**Fig. 2:**
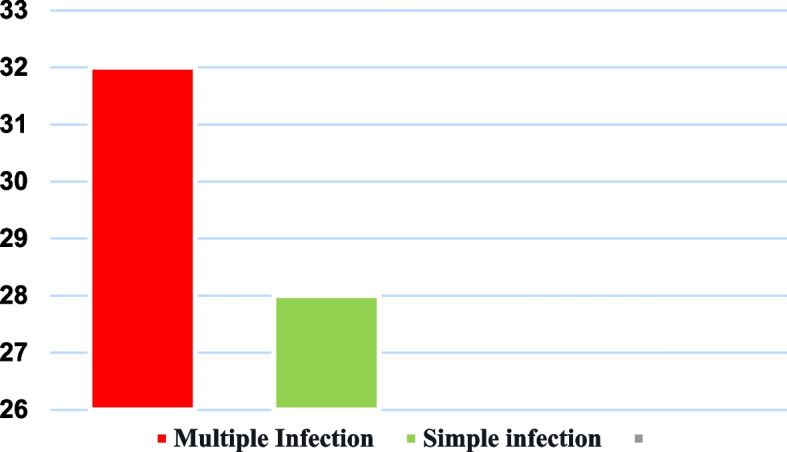
Percentage distribution of women in the sample according to single and multiple HPV infection. Caxias, MA, Brazil, 2022. Source: survey data, 2021/ Legend: Red- Multiple Infection; Green- Simple Infection

In the overall sample, the cases of multiple infections were higher than the cases of single infections. Although multiple infections were more frequent among women than single infections, there was a difference of 12.5% (Fig. 2).

**Fig. 3:**
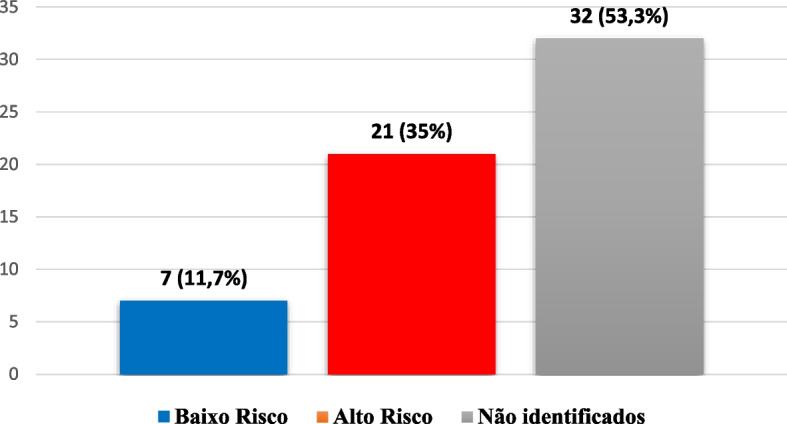
Distribution of women by HPV risk. Caxias, MA, Brazil, 2022. Source: survey data, 2021/ Legend: 1º low-risk; 2º high-risk; 3º unclassified

Of the 28 women in the sample who had simple infections, 35% (*n* = 21) had high-risk HPV and 11.7% (*n* = 07) had low-risk HPV. As shown in Fig. 3, HPV genotypes were not identified in 32 (53.3%) of the cases because It were multiple infections.

**Fig. 4:**
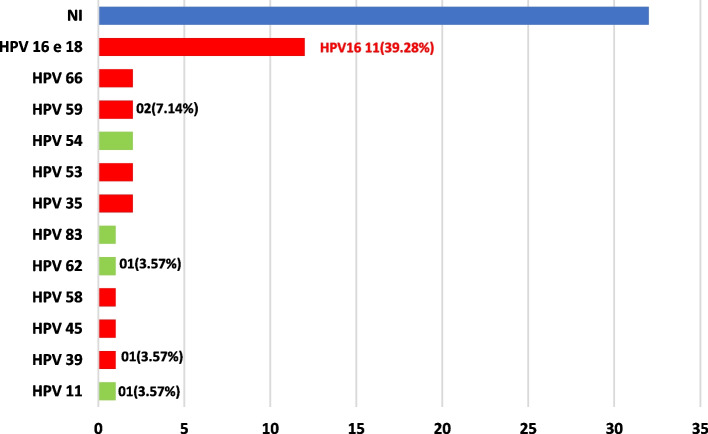
Types of HPV and distribution by risk in Caxias (MA) women. Caxias, MA, Brazil, 2022. Source: survey data, 2021

In the sample 21(35%) high-risk and 11.7% (*n* = 07) low-risk genotypes were identified. The HPV genotypes with the highest frequency was HPV-16 with 39.28% (*n* = 11) cases. The high-risk HPVs identified were: (HPV-16, HPV-18, HPV-66, HPV-56, HPV-59, HPV-58, HPV-53, HPV45, HPV-35, HPV-39) (Fig. 4). 100% (*n* = 09) cases of cervical atypia were infected with HPV, 55% (*n* = 5) were high-risk HPV cases 11.11% (*n* = 01) were low-risk HPV cases and, 33.33% (n = 03) were coinfection cases. 85% (*n* = 51) of HPV infected women had no cervical pap smears. Of these women, 31.37% (*n* = 16) had high-risk HPV, 9.80% (*n* = 05) had low-risk HPV, and 58.82% (*n* = 30) had multiple infections.

## Discussion

Considering the objectives of this research, we summarize its main findings, which are presented below. The sample was characterized in its majority were women living below the poverty line and in essence represented by black women, semi-literate, married and with sexual behavior at risk for HPV infection. The frequency of HPV was high with 41.37% (*n* = 60) positivity in the sample. The rate of cytological atypia was 6.17% (*n* = 9). The frequency of HPV in the quilombola group was higher than in the gypsy women. The multiple HPV infections was higher than the cases of single infections. High-risk HPV infections were more frequent than low-risk HPV infections. All cases of cervical atypia were HPV infected, with the majority being high-risk HPV. Most of the HPV-infected women do not have cervical atypia. A higher frequency of high-risk HPV was identified, especially HPV 16 with 39.28% (*n* = 11) of the cases.

The quilombola communities of Caxias-Ma are located in rural areas of the municipality being distant from the headquarters between 10 and 60 km. It are communities of low socio-economic level that have subsistence farming as their main activity and a large part of its population is registered in the federal government's cash transfer program such as Brazil Aid. The quilombola communities follow a sociocultural pattern where most of them get married at an early age, considering that it is a cultural tradition to form new families and have children. Most of the men work essentially in farming. The quilombola and gypsy areas are poorly assisted by public policies for education and health.

The local gypsy community had its formation process in the 70's due to the migratory movements of the gypsy population coming from other areas of the interior of the state. It is an area of low human development, with a population of socially closed patterns where these people have little access to other areas of the city. For example, marriages are arranged among the people of the gipsy community itself. It survive on diversified commercial activities, and on federal government transfer programs such as Brazil Aid.

The HPV types were not identified when the woman was infected by more than one type because, in these cases, the genome sequencing technique used was of a traditional standard. The cases of multiple infections are sensitive to hybridization techniques and innovative techniques of genome sequencing. Thus, the load of genotypes for multiple infections did not include the HPV frequencies presented here.

HPV performs a functional mission in persistence, regression, and or progression. The rates of regression (60%); persistence (23%); and progression (17%). In HPV 16 and HPV 18 infections there is regression around 50% [10–13]. While HPV infection decreases with age, cancer incidence increases, suggesting that persistence of HPV infection produces high-grade lesions. In 30% to 40% of cases, untreated high-grade cervical intraepithelial neoplasia can progress to invasive cancer [14, 15]. Given the above it is observed the need for follow-up of the 60 cases of HPV infection, in particular, the 35% (*n* = 21) cases of high-risk genotypes without cytological atypia and 09 cases of cytological atypia by HPV primarily in 55% (*n* = 05) cases with high-risk HPV demonstrate these likely conditions of progression revealed.

Women of African descent commonly present cervical cancer in more advanced forms. This fact is more observed due to their greater exposure to adverse social living conditions, as well as precarious accessibility conditions and low supply to health services. Black women have more difficulty participating in organized screening programs for cervical cancer, being exposed to a merely opportunistic and irregular model of screening. In view of the above, we highlight the importance of following up women diagnosed with HPV, especially those at high-risk, considering that It are in conditions of greater social vulnerability [16]. Weber [17] and Backes [18] found a frequency of atypia in their studies carried out in the general population of 3 and 4% respectively, being, therefore, lower in relation to the findings of the research demonstrated. The rates identified in our studies, 4.54% (*n* = 22) gypsy women and 6.50% (*n* = 123) quilombola women being in convergence with this cited evidence.

In the research by Ghosh et al. [19] which used DNA isolation, viral identification and genotyping produced in 441 women with age range less than 30 and greater than 46 years (with mean between 40 and 43 years) in four community tribes in southern India. The prevalence of HPV cases among the tribals was 40.6% (*n* = 179). Being similar to the gypsy group this robust international research showed a higher frequency than our findings of 22.72%.

In the research of Suteu [20] who performed collection with 165 Roma women aged 25–64 years from urban and rural area of Romania in the period from June to November 2015, there was the occurrence of 6% (*n* = 10) cases of atypia where, HPV were present in: ASC-H 8.3% (*n* = 3) and HSIL 2.8% (*n* = 1) cases. Thus in comparison with our findings we observed a lower frequency, in the Roma group (4.54%) but approximate with the identified international research.

In our survey we identified that menopausal women had an increased risk by 4.326 of developing cytological alterations in relation to women who are not in menopause (*p* value 0.045). In the sample used in our research that presented cytological alterations, related to the age range in which these alterations were expressed, the majority of cases occurred between 30 and 64 years with 77.77% (7) cases, and 44.44% of these originated between 50 and 64 years, the age phase in which these women were most exposed to the menopausal phenomenon.

The persistence or recurrence of cervical dysplasia has a major impact on the health quality of life of women. In Borgani's [21] follow-up, 6% of patients (175/2,966) developed persistent/recurrent cervical dysplasia. Median recurrence-free survival was 18 (5–52) months. Overall, 38 (6.9%) patients experienced recurrence. Borgani [21] proposed a nomogram demonstrating the factors affecting the risk of persistence/recurrence of cervical dysplasia. The evaluation criteria are age, body mass index, CIN II and CIN III, other HPV types, academic and non-academic settings, LEEP vs. laser conization, positive ectocervix margins, positive endocervical margins, persistent HPV, and HPV vaccination after conization. The nomogram represents a useful tool to counsel women on their risk of persistence/recurrence after primary conization [21].

The classification of age groups in our research followed the references of Shiffman [22] where the highest frequency of HPV occurred from the age of 10 years with a greater rise at the age of 20 years. This plateau remains until the age of 30. A drop occurs from 30 to 50 years and a stabilization until 70 years. This justified the inclusion in our research of women in the 10 to 64 age group. Most of them did not use and/or used irregular condoms in sexual intercourse varying respectively (89.8% and 95.1%) being more exposed to sexual transmission of HPV.

A research by Lorenzi et al. [23] that evaluated the prevalence of HPV types in women in rural areas over 18 years of age with collection in mobile units in 63 cities of the Hospital de Barretos in the states of Goiânia, Mato Grosso, Minas Gerais and São Paulo women tested positive for HPV (10.0% or *n* = 307). The research was conducted from March to December 2010, using liquid Pap test and HPV genotyping. Since this is also a research in rural women (quilombola group), we observed that the frequency in this group in our research was higher than that detected by the cited author (44.71%—55 cases).

Another robust research conducted in 34 quilombos in 07 cities in MA (São José de Ribamar, Presidente Vargas, Viana, São Luís Gonzaga, Central do Maranhão and Alcântara) with 395 women aged between 12 and 84 years. The most prevalent genotypes were: HPV68 (26%); HPV58; HPV52 (20%); HPV31 (10%) and HPV62 (8%); types were identified: 16, 18, 33, 39, 45, 51, 53, 54, 55, 56, 59, 61, 66, 70, 71, 72, 73, 84. The most prevalent genotypes were HPV68 (26%); HPV58 and HPV52% (20%); HPV31 (10%) and HPV62 (8%) [24]. In comparison we verified divergences because in our research HPV 16 was the most prevalent with 11 (39.28%) cases. The presence of both HPV 16 and HPV 18 was 12 (42.85%). These two types are the most described in the outcomes of cervical cancer cases.

The criteria for defining the grouping of HPV into genus, species and type use the similarity of the genome in the L1 area (most conserved area of the virus). Thus genus share less than 60% similarity. Species share between 60 and 70% similarity and, type (subspecies) shares 71% and 90% similarity. Differences between 1 and 10% and 0.5 and 1% between HPV genome respectively characterize lineage and underline, when it is identical with other similar in 100% HPV [25].

Combination HPV- 16 and 18 antigen therapeutic vaccines as a potential therapeutic in the treatment of gynecological cancers have shown promising results. Most vaccines contain the HPV oncoprotein E6 and E7, and stimulate antigen presentation (APACs). Stimulation leads to the generation of CD8 + cytotoxic T cells or CD4 + helper T cells. Immunotherapies with viral vaccines are based on nucleic acid containing antigens such as HER2/Neu and NY-ESO-1, and although their results were promising, further studies are needed for proof [26–29].

The occurrence of cervical atypia has HPV as a major influencer, however, its occurrence is not related to the cultural behavior of this presenting itself, however, as a null variable. There is no theoretical basis to say that HPV has different occurrence, merely by being a quilombola and/or gypsy community.

The research had limitations considering the sample size of 145 women. The number of 22 Romani women used was also small due to the small population size where the N sample size calculated by statisticians did not allow for the expansion of this sample. As a result of this fact, Romani women were not demonstrated as an ethnic group in most of the variables presented. For future studies it is proposed to expand the number of quilombola communities and the number of women in the two ethnic groups.

## Conclusion

All women with cytological alterations were infected with HPV. There was a high prevalence of HPV infection, higher than the frequencies recorded in Brazil and the Northeast. Most women infected with HPV had multiple infections. There was a predominance of high-risk HPV genotypes and the most frequent was HPV 16.

These findings show that the HPV virus is circulating in these communities and already causing expressions in the cytology exam and with possible short- and long-term repercussions. The data point to the need for further research to identify HPV-16 and high-risk HPV-18 strains and sublines circulating in these unviable populations, as well as the development of effective health policies for long-term follow-up of these cases by the family health strategy.

## Data Availability

The dataset supporting the conclusions of this article is (are) included within the article.
